# Untying the Influence of Advertisements on Consumers Buying Behavior and Brand Loyalty Through Brand Awareness: The Moderating Role of Perceived Quality

**DOI:** 10.3389/fpsyg.2021.803348

**Published:** 2022-01-27

**Authors:** Jin Zhao, Rehan Sohail Butt, Majid Murad, Farhan Mirza, Mamdouh AbdulAziz Saleh Al-Faryan

**Affiliations:** ^1^School of Finance, Shanghai Lixin University of Accounting and Finance, Shanghai, China; ^2^School of Management, Jiangsu University, Zhenjiang, China; ^3^KUBEAC Department, University of Management and Technology, Sialkot, Pakistan; ^4^Department of Accounting and Financial Management, University of Portsmouth, Portsmouth, United Kingdom

**Keywords:** advertisement, brand awareness, brand loyalty, consumer buying behavior, perceived quality

## Abstract

Consumer buying behavior is an important aspect in every marketing strategy to produce maximum output from the market. This study aims to determine how advertisement affects consumer buying behavior and brand loyalty by considering a mediator between brand awareness and the moderating role of perceived quality. For this purpose, this study targets the rising cosmetics industry. This study used the purposive sampling technique to collect data from 300 respondents with the help of an online survey method *via* Google doc. The partial least squares structural equation modeling PLS-SEM was applied to verify the hypotheses relationships. The findings have confirmed that advertisements substantially predicted brand awareness, brand loyalty, and consumer buying behavior. Furthermore, brand awareness partially mediated the association of advertisement with brand loyalty and consumer buying behavior. Also, perceived quality is significantly moderated on the association of brand awareness with brand loyalty and consumer buying behavior. Based on such findings, this study has contributed to the literature and provided new insights into the practical implications alongside the future roadmap of the survey.

## Introduction

Fashion Trends is changing rapidly in the international market (Hur and Cassidy, 2019). Consumers are becoming increasingly brand conscious, and they value branded products to express their status symbol (Turunen and Pöyry, 2019). The consumer desires fashion items that are like their culture. Brittian et al. (2013) found that women have a higher desire to use branded products compared to men. Naturally, the human being is always looking for unique and innovative things. Before brand awareness, women used to wear whatever was available to them (Wei and Lu, 2013). Dörnyei (2020) showed that the emotions of having a unique product help the marketers establish market share by providing exceptional brand elements.

Furthermore, Oh et al. (2020) proposed that the word brand is not a new concept in marketing, rather in the modern era, it is a term exclusively used in the fashion industry. Nettelhorst et al. (2020) explained that marketers changed their mentality from what they want to what their customers want. The brand is an important asset for any business in our local setup because it can change people’s buying behavior. It can play a crucial role in enlarging any business (Choi et al., 2017). There is fierce competition among companies to get a large market share. Rehman et al. (2017) demonstrated that it is very difficult for a company to differentiate its product when many competitors have similar attributes to their product. Jung et al. (2020) discussed why people agree to buy clothes at higher prices. The study found that the consumer’s thinking gets modified.

Similarly, Fazal-e-Hasan et al. (2018) showed that brands were considered highly valuable and helpful in building a relationship with customers. Scholz and Smith (2019) argued that a company’s financial aspect emphasizes the brand’s total value and grows successfully to serve the market. In the current globalizing and emerging markets age, business war depends on price and loyalty, attraction, and related matters (Kim et al., 2019). Alalwan (2018) explained that impressive brand awareness attracts the consumer’s attention and insists they purchase again and again, which results in an increase in sales for a company.

Brand loyalty, brand image, psychical quality and top-of-the-mind brand, and brand recall are ways to measure brand awareness (Sürücü et al., 2019). In the past, women did use expensive items, but the word branded was not clear. These expensive cosmetic items are included in luxuries, but no brand name was used (Çifci et al., 2016). Historically, men and women were not involved in brands too much because of price constraints and their mindset. Through the opportunity to avoid it an early age, young and working-class individuals now confront it as brand slaves (Han et al., 2015). They are always in search of unique designs and better quality. Chung et al. (2017) explained that the quality of any product is judged by price, which is the main reason for satisfaction and dissatisfaction. Before customers buy any brand, they do a lot of research.

Akrout and Nagy (2018) described quality as a key aspect in achieving a company’s wants and business success to grab a place in the global market. Priya et al. (2010) demonstrated that women are the most exclusive consumer for their direct purchase of 80% of total product sold. All types of consumers are highly affected by television advertisements. Nam et al. (2017) discussed how to search for information about brands, one requires internal and external data. Kim and Moon (2020) explained that advertisement and experience are a type of internal information. The data collected through the marketplace, peers, and family is external. The advertisement directly influences consumer awareness, which affects customer loyalty and consumer buying behavior, specifically in the fashion industry.

This study examines the functions of advertisement in building company success and its effect on consumers’ buying behavior and brand loyalty. The aim is to know how the brand is perceived, especially the buying behavior of young men and women. To reinstate a product as top-of-the-mind for consumers, organizations from all areas of the world spend huge amounts on advertisement (Zhao and Yan, 2020). Given its effect on the sales and purchasing behavior of the organization, businesses are unable to decide how to make the most of their advertising and advertising communication (Bagnied et al., 2020). Looking into prior studies, most of the researchers have examined the relationship between consumer purchase intention and social media advertisement (Weismueller et al., 2020), personal factors of consumer buying behaviors (Rehman et al., 2017), brand equity, brand association, and brand awareness on customer buying intentions (Shanahan et al., 2019), social media advertising and customer purchase intention (Alalwan, 2018), and brand awareness, image, physical quality, and employee behavior (Sürücü et al., 2019) in the context of Western economies. Few empirical studies have investigated the impact of advertisement, brand awareness, brand loyalty, perceived quality, and consumer buying behavior in the context of developing countries (Rehman et al., 2017; Rahman, 2018; Shareef et al., 2019). Therefore, to fill this research gap this study is conducted to measure the influence of advertisement on consumer buying behavior and brand loyalty and mediation of brand awareness in this relationship. In addition, it also explores the impact of brand awareness on consumer buying behavior and brand loyalty and the moderation of quality on their relationship. Therefore, this study has proposed the following research questions:

RQ1: What is the influence of advertisement on brand awareness, brand loyalty, and consumer buying behavior?

RQ2: Does brand awareness mediate the relationship between advertisement, brand loyalty, and consumer buying behavior?

RQ3: Does quality moderate the relationship between advertisement, brand loyalty, and consumer buying behavior?

## Literature Review and Theoretical Support

### Theoretical Support

This study used the theory of reasoned action to support this conceptual model. This theory is proposed by Ajzen (1991). According to this theory, attitude toward behavior is one of the important predictors of behavioral intention (Madden et al., 2016; Li et al., 2020). Attitude is defined as “an internal evaluation of an object such as [a] branded product.” Kaur and Hundal (2017) established that consumer attitude and behavior toward the advertisement affects consumer exposure, attention, and reaction to the individual advertisement through a variety of cognitive and affective processes. In consumer buying behavior research, attitude toward the advertisement, attitude toward brand loyalty, and brand awareness are commonly used constructs for predicting the effectiveness of marketing communications on different media (Ayanwale et al., 2005; Alalwan, 2018).

### Advertisement

An advertisement is a valuable tool to divert people’s attitudes positively and attract people toward a product (Shareef et al., 2019). Advertisement is a mode of communication marketing through electronic or print media that persuade the customer to continue or adopt some action by paid content (Cheah et al., 2019). According to Sofi et al. (2018), it is a non-personal way of sharing information related to a product produced by a sponsor with the help of media. Similarly, Ayanwale et al. (2005) proposed that advertising is a paid, non-personal way in which concepts, products or services, ideas, and information are publicized through media (verbal, visual, and te’t) and identified promoter influence behavior. Zhang X. et al. (2020) described that in a company, to meet communication and marketing objectives, mass media plays a vital role and maximum information is provided to the target market about the product. Rehman et al. (2017) purported that the aim of advertising has popularity worldwide. Most companies are spending large amounts of money on advertisement to attract the customer to their products and services. Lichtenthal et al. (2006) summed up that such advertisement is a picture representing the whole story or in written form that the viewer cannot ignore, and it is beneficial for many advertising media.

Fennis and Stroebe (2020) identified that advertising is a promotional marketing strategy to attract people to a product or service. People are in favor of those brands with which they resonate emotionally. The medium can be chosen by your own choice. Some of the mediums are T.V., social media, magazines, and outdoor advertisements.

1. T.V. is the fastest medium of telecommunication for receiving and transmitting multi-colored images and pictures seen by people throughout the world regularly (Masui et al., 2020).
2. Social Media: Most commonly used by the customers, marketers target their customers by posting links on social sites (Zhou et al., 2021).
3. Magazines: Lee and Rim (2017) found that magazine advertisement has a huge impact on customers’ decision-making as the reader is interested in the magazine and forms a relationship with it.
4. Outdoor Advertisement: It includes billboards, posters, broachers, and banners (Weismueller et al., 2020).

#### Repeated Exposure

Repetition of advertising increases product exposure to increase customer popularity (Cox and Cox, 2017). In previous studies, researchers assessed that the repeating and selection of advertising methods should be in accordance with the product categorization, brand positioning, format, and advertising goals (Green et al., 2008; Montoya and Horton, 2020). Prior studies have identified the various impacts of repeated advertising and supporting advertising appeals on brand purchase intention for distinct product classes (Belanche et al., 2017; Wang et al., 2017). More exposure to advertising repetition develops a favorable customer mindset. As a consequence, it is more effective to repeat announcements of known goods compared to announcements of new ones (Yang, 2018). Repeating ads enables marketers to inform customers about the goods and familiarize them with an advertised brand, which improves the likelihood of the products being purchased indirectly. Repeated exposure to advertising has a beneficial impact on customers’ purchasing decisions and helps them remember the brand’s goods (Montoya et al., 2017).

#### Celebrity Endorsement

Advertisers also use celebrity endorsements in their advertisements to sway customer attitudes (Gilal et al., 2020). Celebrities are people who are well-known among the general public for reasons other than their support of certain brands or goods (Schimmelpfennig and Hunt, 2020). Advertisers’ employment of celebrity has a great impact on consumers’ attitudes about advertising (Osei-Frimpong et al., 2019). Only when a well-known celebrity is supporting an advertisement will people buy the goods, regardless of whether or not they know anything about them. Popular celebrity endorsement affects buying intention more than unknown celebrity endorsement (Yang, 2018). According to the experts who conducted the experiments cited above, celebrity endorsements have a favorable effect on customers’ purchasing intentions (Zhang X. et al., 2020).

#### Sexual Appeals

Marketers’ goal was to make the commercial more glamorous and enticing to persuade customers to buy the goods by pushing its picture in their minds (Wirtz et al., 2017). As a result, customers are more likely to buy the goods because of the advertisement’s sexual attractions. When words alone are not doing the job, sex appeal is often utilized in commercials to draw customers’ intention (Black and Morton, 2015). Sexual appeal in advertising has a greater impact on women’s purchasing decisions and self-esteem. Using sexually explicit advertising reduces customers’ desire for product knowledge while increasing efficiency by influencing their purchasing decisions (Gong and Shurtliff, 2020). The advertisements featuring attractive models attracted customers of the opposite sex and impacted their purchasing decisions (Ekici et al., 2020). More and more image-based advertisements include sexual themes, and cosmetic product advertising is a good place for testing the impact of these themes on consumer advertising attitudes (Vargas-Bianchi and Mensa, 2020).

### Consumer Buying Behavior

Consumer behavior involves making a purchase decision based on available resources, i.e., effort, money, and time (Chiang et al., 2016). Furthermore, Tsao et al. (2019) proposed a holistic view of consumer buying behavior. Consumer behaviors are those activities and processes in which individuals choose and utilize ideas, products, services, and experiences. Li et al. (2021) stated that consumer behavior analysis is another tool to examine the complexity of marketing operations. Meanwhile, Sumi and Kabir (2018) demonstrated that today’s consumers are kept in the dark about when and what they desire, all of which results in interactive advertising. Consumer behavior is a mixture of consuming and purchasing products and services (Sundararaj and Rejeesh, 2021). Therefore, Anetoh et al. (2020) explored seven steps of consumer buying decision which needs recognition: search for information, pre-purchase, evaluation, purchase, consumption, post-consumption evaluation, and divestment.

### Brand Loyalty

Brand loyalty describes a client’s connection with a brand (Coelho et al., 2018). Brand loyalty is the tendency to be loyal to a brand, and loyalty demonstrates the consumer’s buying intention (Atulkar, 2020). Additionally, Zhang X. et al. (2020) stated that a loyal consumer characterizes a basis for a price premium, a barrier to entry, protection against deleterious price accomplishment, and responding to competitors. The basic dimension of brand equity is brand loyalty. Similarly, the objective of brand management is brand loyalty. If the company needs to examine the strength and weaknesses of its consumer loyalty, whether the consumer is promoting its product more compared to competitors can be examined (Coelho et al., 2019). Moreover, it is the attitude of the consumer on brand preferences from prior shopping experiences of a product summed up (Bairrada et al., 2018). Furthermore, attitudinal loyalty is the degree of dispositional guarantees for some preferences linked with the brand whereas behavioral loyalty is the repeated buying intention of a consumer (Diallo et al., 2020).

### Brand Awareness

Brand awareness plays a significant role in creating consumer buying decisions by bringing three benefits: learning, consideration, and choice (Foroudi, 2019). Sürücü et al. (2019) designate that brand awareness might be known by thickness and deepness. Thickness expresses how easily a brand name will arise in the customer’s mind while purchasing a product. Deepness means how quickly a customer identifies or recalls a brand. Brand awareness will be greater if a product at once possesses both brand thickness and brand deepness; customers will have thought of a definite product when they need to purchase a product (Romaniuk et al., 2017). Furthermore, the brand name is the most vital part. Brand recall and brand recognition are the components of brand awareness. Brand recall means the customer can recall a brand name accurately when they see a product, and brand recognition means the capability of a customer to detect a brand whenever there is a brand sign (Cheung et al., 2019). Brand awareness is a customer’s capability to recall or memorize brand information (Romaniuk et al., 2017). Any product or service variation in the buying behavior is due to brand awareness related to any good or service.

### Perceived Quality

This quality is possessed by an entity capable of specific or indirect desires (Yang et al., 2019). Among handlers, it is the indication of the assured attributes in a product that create pleasure or frustration (García-Fernández et al., 2018). Konuk (2018) express the quality of a product based on the foundation of performance, strength, consistency, advantages, and technology. It is based on consumers’ judgment and experience. Wang et al. (2020) explain the close link between product and service quality, company profitability, and customer gratification. The assessment of the benefits and strength of the client is service product quality. The chief aim of a lot of investigators is perceived quality (Chi et al., 2020). Pooya et al. (2020) determine that perceived quality describes the buyer’s individual quality decisions about a brand’s whole fineness or advantage. The important element of consumers’ preferences and attitudes is the perceived quality, which is a significant issue in defining affective commitment.

## Hypotheses Development

### Advertisement and Consumer Buying Behavior

Advertisement is a source that convinces people to purchase the product at least once in their lives. Celebrities or personas used in ads may positively influence peoples’ buying intention (Shanahan et al., 2019). Consumer buying behavior should be referred to as the choice to buy a product (Sundararaj and Rejeesh, 2021). Advertisers are adapting different techniques to create purchase decisions through effective commercial messages. Additionally, market advertisers use celebrities in commercials to sponsor their product image (Alalwan, 2018). The involvement of celebrities affects the buying intention of the consumer. This study shows that advertisements have a positive effect on consumer buying intention.

Consequently, Vargas-Bianchi and Mensa (2020) remarked that advertisement has a crucial role in the current age as it is an instrument to build society’s behavior regarding products. Ads help people to get information and make a purchasing decision. People’s psychological, emotional, and behavioral aspects are important while making a purchasing decision (Wirtz et al., 2017). Consumer buying behavior can be predicted by relevant brand awareness in the market (Alalwan, 2018). In conclusion, advertisement has a direct relation with consumer buying behavior. If advertisement increases, it will eventually lead toward an increase in buying intention of the consumer. Therefore, the following hypothesis is proposed:

**H1:** Advertisement substantially predicts consumer buying behavior.

### Advertisement and Brand Loyalty

Nowadays, organizations aim to build strong customer relationships rather than provide only products or services to ensure customer loyalty (Kwon et al., 2020). The process of introducing products to customers, making the product known, and selecting the product agreed upon by customers makes customers loyal to a brand (Balakrishnan et al., 2014). Moreover, Ramaseshan and Stein (2014) explained that the degree of commitment when a customer purchases a product of a special brand is named loyalty. Prior researchers enlightened different factors that affect brand loyalty, but this study reveals five factors: easy usage, quality, brand awareness, brand image, and advertisement (Tidwell et al., 1992; Iglesias et al., 2011; Hoewe and Hatemi, 2016).

Advertisement is one of the essential tools to increase the level of identification. Advertisement is a type of cost. According to Shanahan et al. (2019) it is not a cost if an advertisement lasts for a long period. Besides, every year millions of companies are generating revenue that results in brand loyalty and in making customers loyal to a special brand or firm. Consequently, Casteran et al. (2019) demonstrated that advertisement has a direct impact on brand loyalty. Thus, it is concluded that if advertisement spending is increased, there will be an increase in customer loyalty level. Moreover, the following hypothesis is assumed:

**H2:** Advertisement substantially predicts brand loyalty.

### Advertisement and Brand Awareness

Rahman (2018) has commented that advertisement means attracting potential customers from the market. In contrast, Kanungo and Dutta (1966) have commented that advertisement means communicating with customers. In this regard, it will be essential to state that advertisement means attracting potential and existing customers from the market by creating awareness of the brand, product, or service (Chang and Chang, 2014). Similarly, several prior research studies have stated that brand awareness can be predicted by the active marketing campaign of the brand, such as advertisement and promotional activities (Wang and Yang, 2010; Lee et al., 2017). From this perspective, this study proposed the hypothesis:

**H3:** Advertisement substantially predicts brand awareness.

### Brand Awareness as a Mediator

According to Foroudi (2019), brand awareness is created to sell the product or service to the customer. Sundararaj and Rejeesh (2021) stated that brand awareness is a mandatory element of the overall knowledge system in the mind of the customer - how likely a customer is to recognize the brand under different situations, how frequently the brand name comes into the customer mind, and how much they like the brand. Moreover, Çifci et al. (2016) explored that customer’s ability to remember or recall brand information is called brand awareness. Li et al. (2021) summed up that it supports customers to make the best purchase decision where an exceedingly competitive market exists. Kanungo and Dutta (1966) showed that companies try to better use brand awareness by adapting marketing strategies to create awareness among customers. Cheung et al. (2019) identified that it has two aspects: width and depth. Width represents the outcomes when a customer makes a purchase decision when a brand name comes into their mind, and depth refers to the way customers can recall a brand.

According to Alalwan (2018), when companies establish a new market or product, their core purpose is to focus on creating awareness among customers to get the best results, as brand awareness creates positive brand loyalty. Coelho et al. (2018) explored that brand loyalty is a customer’s past psychological attachment and affection to any brand. It can be measured by taking note of repeated purchases from the same brand. Moreover, Atulkar (2020) examined that to maintain and create a brand, companies must realize the increasing importance of unaided and aided awareness in customers and develop strategies related to it. Market communication should be made with different concerns on public relations and advertisement. Advertising options like radio, television, and social media create awareness.

Zhang H. et al. (2020) explained that a brand’s purchase intention depends upon searching information, problem arousal, comparing alternatives, post-purchase, and purchase behavior. The purchase intention of the customer consists of how much awareness he/she has about a brand. Marketers popularize products with the help of promotional activities to create awareness. When customers use and become aware of any brand, their personal experience will turn into brand loyalty (Sürücü et al., 2019). That effect in purchasing the product again and again in case of a good experience refers to direct loyalty. Thus, we hypothesize:

**H4:** Brand awareness substantially mediates between advertisement and consumer buying behavior.

**H5:** Brand awareness substantially mediates between advertisement and brand loyalty.

### Perceived Quality as a Moderator

In this section, this study discusses the modifying aspect of quality on the association of brand awareness and consumer buying behavior. There is an important relationship between brand awareness and perceived quality (García-Fernández et al., 2018). Few researchers have explored the moderating role of perceived quality on the relationship between brand awareness and consumer buying behavior. It is further suggested that when the brand awareness is high, customer quality evaluation is also high (Yang, 2018). In addition, Wang et al. (2020) explored that perceived quality will affect consumer buying intention and that quality will positively influence purchase intention.

Li et al. (2021) assert that a highly well-known brand will have a greater purchase desire than a less well-known brand. Furthermore, prior studies remarked that perceived quality and purchase intention are positively correlated (Sürücü et al., 2019; Yang et al., 2019). Thus, there is a direct relationship between brand awareness and quality. Romaniuk et al. (2017) described that brand awareness has a significant and positive relationship with quality. Therefore, previous studies argued that higher awareness results in higher perceived quality (Sürücü et al., 2019; Chi et al., 2020). Thus, the following hypothesis is predicted:

**H6:** Perceived quality substantially moderates the relationship between brand awareness and consumer buying behavior.

In this section, perceived quality has a moderating effect on the relationship between brand awareness and brand loyalty. Konuk (2018) explained that perceived quality is related to emotional value. Zhang H. et al. (2020) explained that the road map to brand loyalty is perceived quality. Moreover, Chang and Chang (2014) describes that brand quality is a limitation to measure brand excellence. Furthermore, Yang et al. (2019) elaborated that different people have different perspectives on the same product; when evaluating a product, their attitudes, values, and experiences are considered. Their attitude toward the product is important to measure quality, and feedback is obtained from people who use the product to assess the brand’s quality. Prior studies show that perceived quality will influence brand loyalty and trust and affect purchase behavior (Pooya et al., 2020). Thus, perceived quality and brand loyalty are significantly and positively correlated, and brand loyalty will increase if the perceived quality is increased.

**H7:** Perceived quality substantially moderates the relationship between brand awareness and brand loyalty.

### Theoretical Model

**FIGURE 1:**
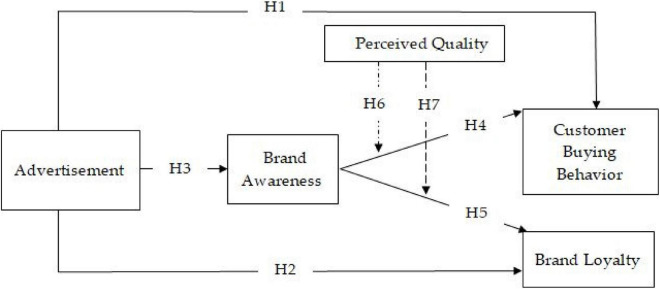
Conceptual model.

To identify the impact of advertisement on consumer buying behavior and brand loyalty, as well as the mediating role of brand awareness and moderating influence of quality, we have conceptualized this theoretical model. Figure 1 shows the research model for consumer buying behavior and brand loyalty.

## Methodology

The current study aims to determine how advertisement affects consumer buying behavior and brand loyalty by considering a mediating role of brand awareness and the moderating role of perceived quality. This study is quantitative and descriptive. However, this study followed deductive reasoning because the foundations of the study are linked with existing literature. Similarly, this study followed a cross-sectional design to gather data from respondents. A questionnaire survey technique was implemented to attain the online feedback of customer responses by using the purposive sampling technique.

### Data Collection Procedure

The target population of the study was consumers of cosmetics brands. Therefore, this study has developed an online questionnaire by using Google docs. The link of the questionnaire has been spread over different social media platforms to gather responses. From this perspective, it can be stated that the present study has followed the purposive sampling method because it allows researchers to request respondents to spread the link to the questionnaire. When the responses of the questionnaire reached 328, the study compiled data in the SPSS file. However, twenty-eight questionnaires consist of empty responses and are considered invalid. Therefore, this study has employed analysis on the valid responses, which are 300 responses with a participation rate of 91%.

### Measures

All the measures were adapted from earlier valid and reliable scales (See Appendix here). To measure the items, a 5-point Likert scale (5 demonstrating “strongly agree,” 4 signifying “agree,” 3 signifying “neutral,” 2 signifying “disagree,” and 1 demonstrating “strongly disagree”) was used.

#### Advertisement

The brand advertisement was measured using three dimensions, namely repeated exposure, celebrity endorsement, and sex appeal, adapted from the study of Kaur and Hundal (2017). Each item has three measurement constructs. A sample item for repeated exposure is “repetition makes me remember the ad.” A sample item for celebrity endorsement is “products endorsed increases the loyalty of the customers.” A sample item for sexual appeal “sex appeal make the ad more attractive and attention-seeking.”

#### Brand Awareness

Brand awareness was assessed using a five-item scale adapted from the study of Sasmita and Mohd Suki (2015). This scale was tested and validated by prior researchers (Foroudi, 2019). A sample item is “I know how this particular product/brand looks.”

#### Brand Loyalty

Brand loyalty was measured using a three items scale and adapted from the study of Sürücü et al. (2019). This scale was widely accepted and used by previous researchers in the field of marketing (Zhang S. et al., 2020). A sample item is “this brand would be my first choice.”

#### Consumer Buying Behavior

To measure consumer buying behavior, we adapted four items scale from the study of Sürücü et al. (2019). This scale was tested and verified by existing studies (Li et al., 2021). A sample item is “I mostly buy luxury brand goods for myself.”

#### Perceived Quality

Perceived quality was measured using a five items scale and adapted from the study of Shanahan et al. (2019). A sample item is “this brand is of high quality.”

### Profile of the Respondents

Table 1 show that most respondents were among the age group of 20–25, with a percentage of 79.3%. A further 13.7%, 4.7%, 1%, and 1% were from the age groups of 20–25, 26–30, 31–35, 36–40, and 40+, respectively. Regarding education, 1.3, 11, 51, 23.3, and 13.3% of respondents belonged to matric, intermediate, bachelors, masters, and MS/M.Phil., respectively. Likewise, 19.3, 4.3, 73, and 3.3% of respondents reported themselves as employed, unemployed, student, and others, respectively. Additionally, 16.3% were married, and 83.7%were unmarried. Similarly, 9% of respondents were users of MAC and 5.7, 19.3, 3, 7.7, 33.7, and 21.7% of respondents were users of Etude, L’OERAL, Avon, and Nivea, Dove, and others, respectively.

**TABLE 1:** Demographic information.

Description		Frequency and percentage
Gender	Male	105 (35%)
	Female	195 (65%)
Age	20–25	239 (79.7%)
	26–30	41 (13.7%)
	31–35	14 (4.7%)
	36–40	3 (1%)
	40– Above	3 (1%)
User of brands	MAC	27 (9%)
	Etude	17 (5.7%)
	L’OREAL	58 (19.3%)
	Avon	9 (3%)
	Nivea	23 (7.7%)
	Dove	101 (33.7%)
	Other	65 (21.7%)
Marital status	Single	251 (83.7%)
	Married	49 (16.3%)
Employment status	Employed	58 (19.3%)
	Unemployed	13 (4.3%)
	Students	219 (73%)
	Other	10 (3.3%)
Qualification	Matric	4 (1.3%)
	Intermediate	33 (11%)
	Bachelor	153 (51%)
	Masters	70 (23.3%)
	MS/M.Phil./PhD.	40 (13.3%)

## Results

### Measurement Model

The measurement model was analyzed through reliability and validity. Construct reliability was assessed using Cronbach’s alpha and composite reliability. Table 2 shows the values of Cronbach’s alpha and composite reliability for advertisement (0.888, 0.910), brand awareness (0.926, 0.945), consumer buying behavior (0.895, 0.927), brand loyalty (0.902, 0.939), and perceived quality (0.932, 0.949). According to Hair et al. (2014), the values of Cronbach’s alpha should be >0.70 and the values of composite reliability should be >0.80. Therefore, the values of Cronbach’s alpha and composite reliability were acceptable and above the threshold value (Sarstedt and Cheah, 2019). Moreover, construct validity was analyzed using average variance extracted AVE. The values of AVE were presented in Table 2. The values of AVE for advertisement were (0.530), brand awareness (0.774), consumer buying behavior (0.761), brand loyalty (0.837), and perceived quality (0.787). Thus, all the values of validity fall within the range of the threshold value of 0.50 suggested by Sarstedt et al. (2011). Furthermore, to check the multicollinearity issue, variance inflation test VIF was performed. The values of VIF were also shown in Table 2. According to Hair et al. (2014), the values of VIF must be lower than 5. Hence, the entire construct’s VIF were under the threshold value and there is no issue of multicollinearity in the data.

**TABLE 2:** Measurement model.

Variable and constructs	Loadings	α	CR	AVE	VIF
**Advertisement**		**0.888**	**0.910**	**0.530**	
ADV1	0.825				4.454
ADV2	0.805				4.790
ADV3	0.793				3.924
ADV4	0.755				2.298
ADV5	0.756				2.652
ADV6	0.743				2.704
ADV7	0.737				3.120
ADV8	0.790				3.380
ADV9	0.724				3.499
**Brand awareness**		**0.926**	**0.945**	**0.774**	
BRA1	0.863				2.889
BRA2	0.790				2.175
BRA3	0.884				3.217
BRA4	0.934				4.864
BRA5	0.919				4.319
**Consumer buying behavior**		**0.895**	**0.927**	**0.761**	
CBB1	0.830				2.078
CBB2	0.883				2.668
CBB3	0.893				2.857
CBB4	0.883				2.669
**Brand loyalty**		**0.902**	**0.939**	**0.837**	
BRL1	0.926				3.183
BRL2	0.917				3.128
BRL3	0.900				2.499
**Perceived quality**		**0.932**	**0.949**	**0.787**	
PRQ1	0.908				4.111
PRQ2	0.892				3.464
PRQ3	0.861				2.702
PRQ4	0.860				2.515
PRQ5	0.912				4.269

*α, Cronbach’s alpha; CR, composite reliability; AVE, average variance extracted; VIF, variance inflation factor.*

### Discriminant Validity

Discriminant validity test was assessed using both criteria’s Fornell and Larcker (2018a) and Heterotrait-Monotrait HTMT ratio. The findings were shown in Tables 3, 4. As per criteria (Fornell and Larcker, 2018b), the square root of the AVE is called discriminant validity and must be higher than correlations values. Moreover, the values of the HTMT ratio should be less than 0.85. Thus, it is seen that the maximum achieved HTMT value was 0.599, and below the threshold value as suggested by Sarstedt et al. (2011). Thus, all the measurement constructs were acceptable for structural model analysis.

**TABLE 3:** Fornell-Larcker criterion.

	ADV	BRA	BRL	CBB	PRQ
ADV	**0.728**				
BRA	0.486	**0.880**			
BRL	0.538	0.427	**0.915**		
CBB	0.489	0.331	0.413	**0.873**	
PRQ	0.268	0.344	0.360	0.363	**0.887**

*items with diagonals are the square root of the AVE. Items under diagonals are the correlations. ADV, advertisement; BRA, brand awareness; CBB, consumer buying behavior; BRL, brand loyalty; PRQ, perceived quality.*

**TABLE 4:** Heterotrait-Monotrait ratio (HTMT) criterion.

	ADV	BRA	BRL	CBB	PRQ
ADV					
BRA	0.534				
BRL	0.599	0.465			
CBB	0.550	0.359	0.458		
PRQ	0.292	0.369	0.391	0.395	

*ADV, advertisement; BA, brand awareness; CBB, consumer buying behavior; BL, brand loyalty; PRQ, perceived quality.*

### Structural Model

**FIGURE 2:**
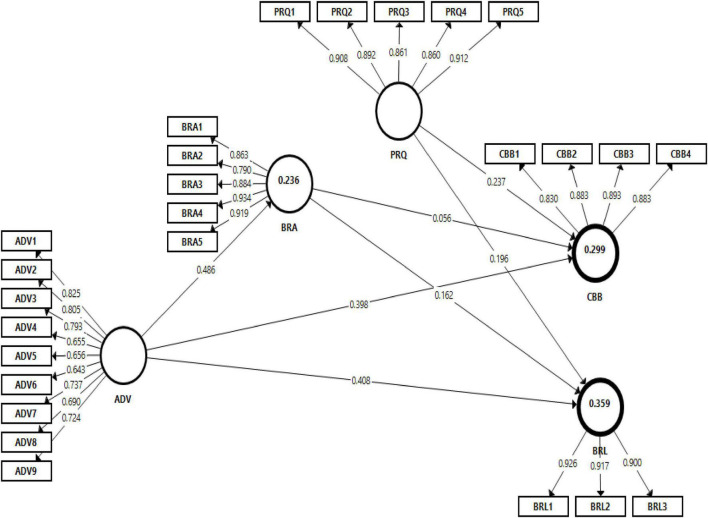
Structural model.

The structural model was analyzed through Smart-PLS software and partial least squares structural equation modeling technique PLS-SEM was performed using the bootstrap method with 5000 sub-samples. This software was widely used and accepted in the field of management and social sciences studies (Vinzi et al., 2010; Hair et al., 2014; Sarstedt et al., 2014a; Cai et al., 2021). The fitness of the structural model was assessed through the standardized root mean square residual SRMR value. According to Sarstedt, Ringle, and Sarstedt et al. (2014b) a good structural model must have <0.080 SRMR value. Therefore, the value of SRMR was 0.070, which indicates an acceptable and adequate level of structural model fitness. Moreover, the structural model was also assessed using the value of the determination coefficient *R*^2^. As suggested by Chin (2010), the desired *R*^2^ should be greater than 0.1 or zero. Table 5 and Figure 2 shows that the structural model explained 23.6% variance in brand awareness, 29.9% in consumer buying behavior, and 35.9% in brand loyalty. Consequently, the values of *R*^2^ were acceptable.

**TABLE 5:** Strength of model.

	*R* square	*R* square adjusted
BRA	0.236	0.234
BRL	0.359	0.355
CBB	0.299	0.295

*BRA, brand awareness; CBB, consumer buying behavior; BRL, brand loyalty.*

Additionally, for the predictive relevance of the model, the cross-validated redundancy measure (blindfolding) *Q*^2^ test was performed. According to Götz et al. (2010), the value of *Q*^2^ must be >0.1 or zero. Table 6 explains that the values of *Q*^2^ exceeded 0.1 and the positive predictive significance level of the model.

**TABLE 6:** Cross-validated redundancy.

	SSO	SSE	*Q*^2^ (=1-SSE/SSO)
BRA	2430.000	1991.731	0.180
BRL	1458.000	1031.349	0.293
CBB	1944.000	1511.844	0.222

*BRA, brand awareness; CBB, consumer buying behavior; BRL, brand loyalty.*

### Testing of Hypothesis

**FIGURE 3:**
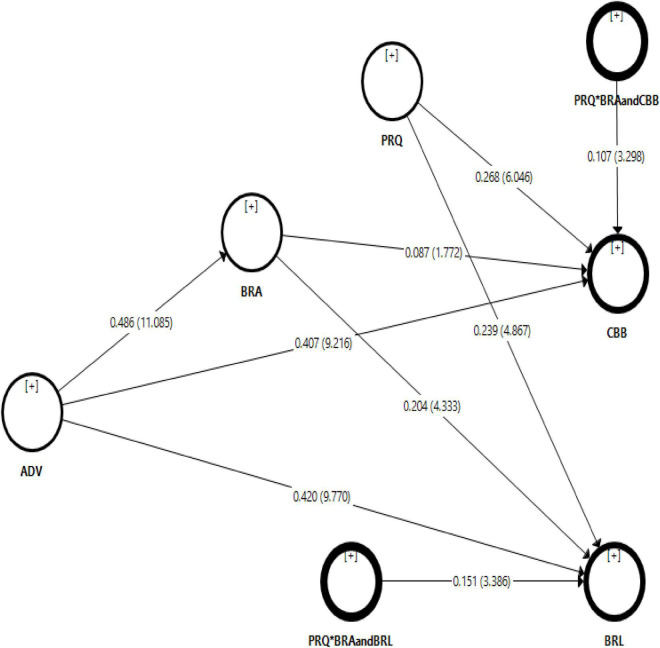
Bootstrapping.

The results of the hypotheses were presented in Table 7 and Figure 3. To test hypothesis H1, findings show that advertisement has a positive and significant impact on consumer buying behavior (β = 0.407, C.R = 9.216, *p* < 0.000). It means that if there is a more attractive advertisement about the brand, it will ultimately increase customers’ buying behavior. Therefore, H1 was accepted. Moreover, H2 results illustrate that advertisement has a positive and significant influence on brand loyalty (β = 0.420, C.R = 9.770, *p* < 0.000). Increased advertisement creates more brand loyalty among customers to satisfy their needs. Hence, H2 was supported. Moreover, H3 results indicate that advertisement has a positive and significant impact on brand awareness (β = 0.486, C.R = 11.085, *p* < 0.000). Hence, H3 was accepted. Furthermore, findings show that brand awareness has a positive and significant impact on consumer buying behavior (β = 0.087, C.R = 1.772, *p* < 0.047) and brand loyalty (β = 0.204, C.R = 4.333, *p* < 0.000). Additionally, this study also hypothesized that brand awareness plays a mediating role (indirect effect) on the relationship between advertisement, consumer buying behavior, and brand loyalty. The H4 findings illustrate that brand awareness positively and significantly mediates the relationship between advertisement and consumer buying behavior (β = 0.042, C.R = 1.723, *p* < 0.046). Therefore, H4 was accepted. Moreover, H5 results show that brand awareness positively and significantly mediates the relationship between advertisement and brand loyalty (β = 0.099, C.R = 3.801, *p* < 0.000). Thus, H5 was also supported.

**TABLE 7:** Structural model path coefficients.

	Relationships	Original sample (O)	Sample mean (M)	Standard deviation (STDEV)	*T* statistics (| O/STDEV|)	*P* values
	**Direct effects**					
H1	ADV → BRA	0.486	0.487	0.044	11.085	**0.000**
H2	ADV → BRL	0.420	0.417	0.043	9.770	**0.000**
H3	ADV → CBB	0.407	0.405	0.044	9.216	**0.000**
-	BRA → BRL	0.204	0.209	0.047	4.333	**0.000**
-	BRA → CBB	0.087	0.088	0.049	1.772	**0.047**
	**Indirect effects**					
H4	BRA → ADV → CBB	0.042	0.043	0.024	1.723	0.046
H5	BRA → ADV → BRL	0.099	0.102	0.026	3.801	0.000

*ADV, advertisement; BRA, brand awareness; CBB, consumer buying behavior; BRL, brand loyalty; PRQ, perceived quality. Items with diagonals are indicated in bold.*

### Moderation Analysis

To assess the moderating role of perceived quality in the relationship between brand awareness and consumer buying behavior, Table 8 results show that perceived quality has a positive influence on consumer buying behavior (β = 0.268, C.R = 6.046, *p* < 0.000) and also positively moderates the relationship between brand awareness and consumer buying behavior (β = 0.151, C.R = 3.386, *p* < 0.001). So, H6 was accepted. Meanwhile, H7 findings indicate that perceived quality has a significant impact on brand loyalty (β = 0.239, C.R = 4.867, *p* < 0.000) and significantly moderates the relationship between brand awareness and brand loyalty (β = 0.107, C.R = 3.298, *p* < 0.001). Thus, H7 was also supported.

**TABLE 8:** Moderation analysis.

	Moderating effects					
-	PRQ → CBB	0.268	0.269	0.044	6.046	0.000
-	PRQ → BRL	0.239	0.235	0.049	4.867	0.000
H6	PRQ * BRA and CBB	0.151	0.144	0.044	3.386	0.001
H7	PRQ * BRA and BRL	0.107	0.110	0.033	3.298	0.001

*BRA, brand awareness; CBB, consumer buying behavior; BRL, brand loyalty; PRQ, perceived quality.*

## Discussion

This study aims to determine how advertisement affects consumer buying behavior and brand loyalty by considering a mediator between brand awareness and the moderating role of perceived quality. The study’s findings have revealed that advertising substantially predicted consumer behavior while brand loyalty mediated it, and perceived quality is moderated on their association. This study has confirmed that buying behavior is substantially predicted by advertisement and brand awareness. Similarly, Foroudi (2019) has confirmed that brand awareness is created by significant marketing campaigns of the companies, such as advertisements. It is also confirmed by the present study that advertisements are substantially linked to brand awareness in the cosmetics branding context. Furthermore, this study has also confirmed that brand awareness is significantly linked with consumer buying behavior. In this regard, Romaniuk et al. (2017) has commented that consumers create variation in their buying pattern due to significant brand awareness. However, several prior research studies have demonstrated that brand awareness attracts consumers toward the product or service and increases potential customers (Kim et al., 2019; Shanahan et al., 2019). From this perspective, this study has concluded that brand awareness created by advertisements influences the buying behavior of cosmetics consumers.

Sofi et al. (2018) stated that advertisement substantially predicts consumer buying behavior, while such an association becomes stronger when advertisement actively produced positive outcomes. In the same sense, this study has proved the mediation effect of brand awareness between advertisement and consumer buying behavior. It implies that consumer buying behavior increases with an increase in an advertisement while such an increment becomes robust when brand association plays an active role. In contrast, this study has also confirmed the mediation effect of brand awareness between the association of advertisement and brand loyalty. In this regard, several prior research studies have stated that consumers become more loyal toward the brand when brand awareness substantially works (Sasmita and Mohd Suki, 2015; Sürücü et al., 2019). Therefore, this study has concluded that consumers become more loyal and demonstrate constructive buying behavior because of the advertisement, and such association becomes robust based on brand awareness.

Furthermore, this study has found that perceived quality moderated the relationship between brand awareness, brand loyalty, and consumer buying behavior. In this regard, several prior research studies have stated that perceived quality attracts potential consumers from the market, and consequently, the company’s growth increases (Akrout and Nagy, 2018; García-Fernández et al., 2018). However, this study has tested moderation of perceived quality which is statistically supported by the findings. Therefore, it is concluded that brand awareness increases loyalty and buying patterns and that when perceived quality is offered, brand awareness substantially predicts consumer buying behavior and brand loyalty.

### Theoretical and Practical Implications

This study has contributed to the literature by evaluating the moderation effect of perceived quality on brand awareness with loyalty and consumer buying behavior. However, Teo et al. (2019) have confirmed that when a brand offers substantial-quality products and increased awareness in the market, it predicts the consumers’ higher purchasing behavior. It implies that perceived quality can be taken as a moderator. Therefore, this study has considered perceived quality as a moderator and tested empirically. Furthermore, the cosmetics industry is a growing industry worldwide and lacks research attention (Amberg and Fogarassy, 2019). Therefore, this study has focused on the cosmetics industry to analyze the theoretical framework of the study. In this regard, this study has contributed to the literature of the cosmetics industry by stating that young people have a higher intention to demonstrate higher buying behavior. Therefore, managers have to focus on marketing campaigns focused on the younger population to produce a higher market share.

The findings of the study have confirmed that consumers preferred branded cosmetics products because they are more sensitive about their social standards. These consequences have important suggestions for international selling directors. With the increase in the quantity of cosmetics brands, brand managers and selling directors must evolve and understand the promotional activities from the Pakistani point of view. Outcomes would lead cosmetics product brand managers to develop policies to progress their branding decisions to gain a more competitive edge and stability of business through loyal customers. Consequences suggested that managers focus on brand awareness to increase consumer loyalty and consumer buying behavior by using promotional activities like advertisements. Teenagers are spending more time on social media sites like Facebook, Instagram, and Twitter, consequently, it will also be helpful for managers to create awareness in the mind of customers through social media. Meanwhile, to increase loyalty and consumer buying behavior, cosmetics product managers should pay more attention to building trust between their consumers by meeting or going beyond their expectations.

### Limitation and Future Direction

The study’s findings are generalizable to the entire cosmetics industry, although this study has some limitations, just like other studies. For instance, one limit was due to the responses of the questions, which depended upon the Likert-type scale. Some people do not give a careful response, and others like to give careful answers. It means the presenter influenced the respondent’s reaction. Future research could be carried out in other sectors, including the telecom sector, banking sector, and textile sector, to show the cross-sector investigation of c consumer buying behavior and their outcome on performance, and the data should be collected using a mixed approach. Using this, the result might change. In the future, sample size should also be increased. Different promotional tools can be considered for further study to evaluate consumer behavior concerning perceived quality and brand awareness.

## Data Availability Statement

The raw data supporting the conclusions of this article will be made available by the authors, without undue reservation.

## Ethics Statement

The studies involving human participants were reviewed and approved by Ethics Committee of the Jiangsu University China. The patients/participants provided their written informed consent to participate in this study.

## Author Contributions

RB and MM proposed the research, analyzed the experimental results, and wrote the manuscript. JZ, FM, and AS designed and carried out the revision of this manuscript and extensively edited the manuscript. All authors contributed to the article and approved the submitted version.

## Conflict of Interest

The authors declare that the research was conducted in the absence of any commercial or financial relationships that could be construed as a potential conflict of interest.

## Publisher’s Note

All claims expressed in this article are solely those of the authors and do not necessarily represent those of their affiliated organizations, or those of the publisher, the editors and the reviewers. Any product that may be evaluated in this article, or claim that may be made by its manufacturer, is not guaranteed or endorsed by the publisher.

## Appendix

**Table:**

	Customer buying behavior
CBB1	I can imagine buying (product) from this company.
CBB2	I am very interested in buying (product) from this company.
CBB3	I mostly buy luxury fashion goods for myself.
CBB4	I mostly buy imported fashion goods for myself.

	**Advertisement**

***Repeated exposure***	
ADV1	Repetition makes me remember the ad and helps in making better choices.
ADV2	First time repetition forms an attitude about new product and makes it familiar.
ADV3	Two and three repetitions have a psychological impact on mind.
***Celebrity endorsement***	
ADV4	Products endorsed by celebrities are of superior quality.
ADV5	Celebrity endorsements increase the loyalty of the consumers.
ADV6	Adding a celebrity in the ad increases the purchase intention.
***Sexual appeal***	
ADV7	Sex appeal makes the ad more attractive and attention seeking.
ADV8	Sex appeals make me willing to pay even more price to buy the product.
ADV9	Sex appeal makes me feel confident after using the advertised product.

	**Brand awareness**

BRA 1	I am aware this particular product/brand appeared in social media.
BRA 2	I can recognize this particular product/brand in comparison with the other competing product/brand that appeared in social media.
BRA 3	I know what this particular product/brand looks like.
BRA 4	Some characteristics of the particular product/brand that appeared in social media come to my mind quickly.
BRA 5	I can quickly recall the symbol or logo of the particular product/brand that appeared in social media.

	**Brand loyalty**

BRL1	I consider myself to be loyal to this brand.
BRL2	This brand would be my first choice.
BRL3	I will not buy other brands if this brand is available at the store.

	**Perceived quality**

PRQ1	This brand is of high quality.
PRQ1	The likely quality of this brand is extremely high.
PRQ1	The likelihood that this brand would be functional is very high
PRQ1	The likelihood this brand is reliable is very high.
PRQ5	This brand must be of very good quality.
